# Modifiable factors predict symptom progression in dementia over eight years

**DOI:** 10.1002/alz70860_097492

**Published:** 2025-12-23

**Authors:** Iris Blotenberg, Felix Wittström, Bernhard Michalowsky, Moritz Platen, Diana Wucherer, Stefan Teipel, Wolfgang Hoffmann, Jochen René Thyrian

**Affiliations:** ^1^ Deutsches Zentrum für Neurodegenerative Erkrankungen e. V. (DZNE), site Rostock / Greifswald, Greifswald, Mecklenburg‐Vorpommern, Germany; ^2^ Karolinska Institute, Stockholm, Sweden; ^3^ Deutsches Zentrum für Neurodegenerative Erkrankungen e. V. (DZNE), site Rostock / Greifswald, Greifswald, Germany; ^4^ Deutsches Zentrum für Neurodegenerative Erkrankungen e. V. (DZNE), site Rostock / Greifswald, Rostock, Germany; ^5^ Department of Psychosomatic Medicine, University Medicine Rostock, Rostock, Germany; ^6^ Institute for Community Medicine / University of Greifswald, Greifswald, Germany; ^7^ Institute for Community Medicine, University Medicine Greifswald, Greifswald, Mecklenburg‐Vorpommern, Germany

## Abstract

**Background:**

The aim of the present study was to investigate the association between modifiable factors and symptom progression in people with dementia over a period of up to eight years.

**Method:**

We used data from a German cohort of community‐dwelling individuals who screened positive for dementia in primary care. They underwent comprehensive annual in‐home‐assessments for up to eight years by specially trained nurses. The following modifiable factors were considered: low education, hearing impairment (and its treatment), hypertension (and its treatment), alcohol consumption, obesity, smoking, depression, social isolation, physical inactivity, diabetes (and its treatment) and visual impairment. We used multilevel growth curve models to investigate the role of modifiable risk factors on cognitive trajectories and trajectories in daily functioning.

**Result:**

Higher education was associated with higher cognitive status at the beginning of the study, but also with faster cognitive decline over time. People receiving anti‐diabetic medications showed slower cognitive decline, while depression and visual impairment were associated with lower levels of daily functioning at baseline and faster cognitive decline over the eight‐year study period. We found no association of hearing impairment (or its treatment), hypertension (or its treatment), alcohol consumption, obesity, smoking, lack of social support and physical inactivity with the rate of symptom progression.

**Conclusion:**

Our study found evidence that several potentially modifiable risk factors influenced symptom progression in dementia over up to eight years. Cognitive reserve through education showed a positive effect, which reversed over time, and depressive symptoms were linked to less favorable progression. Treating comorbidities like diabetes and visual impairment may positively impact dementia symptoms. Modifiable risk factors are promising targets for tertiary prevention and should be explored further.

